# Mental Health Parity in Interventional Psychiatry: Access, Ethical and Policy Implications

**DOI:** 10.1007/s11920-026-01703-2

**Published:** 2026-07-16

**Authors:** Laura Y. Cabrera, Leonard M. Fleck, Alik S. Widge

**Affiliations:** 1 Center for Neural Engineering, Rock Ethics Institute, The Pennsylvania State University, University Park, PA USA; 2 Center for Bioethics and Social Justice, Michigan State University, East Lansing, MI USA; 3 Department of Psychiatry and Behavioral Sciences, University of Minnesota, Minneapolis, MN USA

**Keywords:** Interventional psychiatry, Mental health, Parity, Disparity, Access

## Abstract

**Purpose of Review:**

Mental health disorders are a significant public health problem with profound impacts on individual health, communities and society. Interventional approaches are limited in both utilization and access, despite their strong evidence base in specific disorders. The objective of this review is to describe factors and challenges influencing equity in interventional psychiatry.

**Recent Findings:**

Mental health care inequities are pervasive and persistent. For interventional psychiatry tools, even with current evidence of interventions’ cost-effectiveness, inequities in access persist based on geographic context, socioeconomic status, and race, as well as insurers’ reluctance to cover some of them.

**Summary:**

A wide range of social factors impact access and utilization of interventional psychiatry tools. To ensure parity, we need strategies to increase awareness and to address access and utilization disparities found across these interventions, in addition to keeping up-to-date mental health coverage policies based on new safety and effectiveness evidence.

## Introduction

Affecting over 1 billion people worldwide, mental health disorders such as anxiety and depression are among the most prevalent in all countries and communities, affecting individuals of all ages and income levels. These disorders constitute the second leading reason for long-term disability, contributing to loss of healthy life and driving up health care costs for affected people and families, thereby inflicting substantial economic losses [1, 2]. Mental health disorders disproportionately affect those closer to the margins of our societies, as they are less likely to receive mental health care [3], undermining both individual well-being and broader public health goals. For individuals who have not responded to first-line mental health treatments, and who could benefit from interventional psychiatry [4, 5] these inequities are compounded. Interventional psychiatry is often costly, concentrated in academic or urban medical centers, and unevenly covered by insurance, leaving marginalized populations disproportionately excluded [6, 7].^1^ Stigma, perceived limited evidence of effectiveness, and limited awareness of these types of treatments [3, 6, 8, 9] are also important factors affecting access to interventions.

Equitable access to advanced psychiatric treatments is essential for reducing the substantial and persistent disparities in mental health outcomes across socioeconomic, racial, and geographic lines. Despite the value and potential of interventional therapies, and despite the existence of mental health parity laws, meaningful disparities persist. In practice, inconsistent parity compliance disproportionately affects marginalized race and ethnic backgrounds, who already experience longstanding disparities in mental health care. This review describes the promises and challenges of mental health parity in interventional psychiatry and discusses its ethical and policy implications.

### Mental Health Parity: Legal and Policy Landscape

Around the globe, countries are strengthening their mental health policies and planning. Many have updated their policies and adopted rights-based approaches to promote mental health parity [10]. In the U.S. the Paul Wellstone and Pete Domenici Mental Health Parity and Addiction Equity Act (MHPAEA) of 2008 expanded past parity laws requiring insurers to apply the same treatment limitations to mental health as to medical and surgical care [11–13]. Similar parity principles are being advocated globally, especially in countries developing comprehensive health financing systems [14].

While parity does not require coverage of a specific minimum set of services, the MHPAEA requires that the financial requirements and treatment limitations applicable to mental health benefits to be no more restrictive than the limitations applied to medical and surgical benefits. Plans cannot impose separate limitations that apply only to mental health care [12, 13]. In practical terms, if an insurer does not cap visits, raise medical-necessity thresholds, or impose stricter utilization controls for conditions such as diabetes or heart disease, the insurer cannot do so for depression, anxiety, or substance use disorders. Parity determinations consider financial requirements and treatment limitations, which limit the scope or duration of benefits for treatment. In addition to quantitative treatment limitations (e.g. visit limits) there are also non-quantitative treatment limitations (NQTLs), which are “non-numeral limits on the scope or duration of benefits for treatment (such as preauthorization requirements)” [11, 13].

Initially aimed at large-group plans, MHPAEA’s reach was significantly broadened by the Patient Protection and Affordable Care Act (ACA), which both extended parity to individual and small-group markets and designated behavioral health services as essential health benefits in Marketplace plans. As a result of subsequent rulemaking and state and federal enforcement roles, parity requirements now reach many public and private coverage arrangements, including Medicaid managed care, employer plans, and qualified individual policies.

In further amendments to the Act, Congress has strengthened transparency and enforcement by requiring comparative analyses of the provision of mental health and medical/surgical services and by mandating that many health insurers publicly release machine-readable files with negotiated reimbursement rates for every covered service [13, 15].

### Interventional Psychiatry: Clinical and Cost-Effectiveness Overview

Interventional psychiatry is a subspecialty within psychiatry that applies electro-magnetic energy to modulate activity in key brain circuits [4, 5]. It leverages current understandings of the brain as an electrochemical organ, where dysfunction in specific neural circuits gives rise to specific psychiatric symptoms [5]. Further, it is gaining momentum as more research focuses on brain circuits [16] as evidenced by the recent creation of the interventional psychiatry consortium^2^. These tools address the profound clinical need engendered by severely ill patients and patients whose disorders do not respond to any of the first-line treatments (e.g. treatment resistant disorders or difficult to treat disorders) or when there is a need for rapid symptom relief. Compared to medications, interventional psychiatry tools more directly modulate brain circuits, minimizing side effects. Among interventional psychiatry techniques used in clinical care for neuropsychiatric disorders are:

Electroconvulsive therapy (ECT): a non-implantable technique that induces a seizure under anesthesia, seen as the “gold standard” treatment for disorders such as major depression disorder (MDD) and catatonia [17, 18].

Transcranial Magnetic Stimulation (TMS): also a non-implantable technique that produces focal activation with an electromagnet. It is approved for depression, obsessive-compulsive disorder (OCD); as an aid in short-term smoking cessation; and more recently, for anxiety (co-occurring with depression) [19].

Vagus Nerve Stimulation (VNS): an implanted device that stimulates vagal afferent fibers in the neck to affect downstream brain circuity. VNS has established efficacy, at least 50% of implanted patients [20], in highly resistant treatment resistant depression [21], but its antidepressant effects can take up to a year to fully manifest.

Deep Brain Stimulation (DBS): also involves an implanted device that directly stimulates specific brain regions or white matter tracts [22, 23]. For mental health it has an FDA humanitarian device exemption (HDE) for OCD (indicating that DBS is safe and has probable benefit), but it is still experimental for most psychiatric conditions, including depression [24].

While the tools of interventional psychiatry can effectively treat different neuropsychiatric disorders, treating any given disorder depends on choosing the right treatment target or targets, on identifying which patients might benefit more from a specific intervention compared to another, and choosing which intervention to try first given the different options available (Table 1). Moreover, it depends on access and timely use.

**Table 1:** Key Indications, Efficacy, Safety Profile, Cost, Reimbursement Profiles and Cost-Effectiveness across 4 Interventional Psychiatry Tools

	ECT	TMS	VNS	DBS
Primary indications (FDA approved)	FDA Class II: MDD (“Gold Standard in TRD”) and catatonia; FDA Class III-schizophrenia, schizoaffective disorder, and mania	Major depression (approved after failure of one antidepressant)*, OCD, substance use disorders	TRD (after failure to respond to 4 different antidepressant treatment trials)	FDA HDE for OCD; Investigational for TRD (not FDA approved)
Efficacy	Most effective acute therapy for major depression; 70–90% response rates	Moderate efficacy; 30–64% response rate [25]; less effective than ECT for psychotic or severe TRD	60–70% response rates in long-term TRD [26], although often slow to onset	60–70% response rate in OCD; rates in other indications (e.g. depression) more variable [23], depending on center; primarily investigational for mental health
Safety profile	Common: headache, nausea, short-term memory loss Rare: cognitive and cardiac risks	Common: Mild side effects (headache, discomfort) Rare: risk of seizure	Surgical risks; voice changes, cough, throat discomfort	Surgical risks (bleeding, infection, hardware complications); in addition to risks from stimulation
Cost (typical ranges)	Per session: $1,500–$2,500 Total Course: (10 sessions): $15,000–$25,000	Per session: ~$300 Total course: $6,000–$12,000 (typical)	Implantation cost (surgery + device): Typically, $40,000–$45,000	Implantation cost (surgery + device): $100,000–$200,000 Device cost alone: ~$35,000
Reimbursed by insurance type	Covered by Medicare (Part A or B), and almost always for Medicaid and private insurance in depression; less certain in other disorders.	For depression covered by Medicare with criteria (after ≥ 1 failed antidepressant; 4–6 week course), for Medicaid, often covered but variable, and for private broadly covered but strict prior authorization. For OCD, not covered by Medicare, Medicaid and private rare/variable tiered with fail-first policies.	For depression, Medicare very limited coverage **, for Medicaid and private payors rarely covered.	For OCD –limited coverage with Medicare (HDE), other insurers variable and usually requires multiple appeals [27]. Other psychiatric – No, considered investigational.
Cost-Effectiveness (ICER)	N/A	US $34,999 per QALY (vs. sham), at US $300 per treatment session [28]	£29,995 per QALY gained (0.42 incremental QALY vs. TAU) [29]	OCD (no rechargeable)- US $153,221 per QALY gained (3-year ICER) less cost effective than TAU OCD (rechargeable)- US$70,646 per QALY gained (more cost-effective) [30]

With cost estimates from various sources, including – [30, 31]

* and/or demonstrates an intolerance to psychopharmacological interventions.- https://www.cms.gov/medicare-coverage-database/view/lcd.aspx?LCDId=34998

https://healingmaps.com/cost-of-tms-therapy/

** until recently covered only through Coverage with Evidence Development (CED) when offered in a CMS approved, double-blind, randomized, placebo-controlled trial with a follow-up duration of at least one year- https://www.cms.gov/medicare-coverage-database/view/ncacal-decision-memo.aspx?proposed=N&NCAId=292

ICER - Incremental Cost-Effectiveness Ratio

QALY: Quality-Adjusted Life Year

HDE: Humanitarian Device Exemption

TRD: Treatment Resistance Depression

When considering whether interventional psychiatry therapies should be offered or covered, cost is just one consideration. Other relevant considerations have to do with their evidence for effectiveness, their side effects, the number of in-person sessions required (like in the case of ECT and TMS which might not work for those with less flexible work arrangement, or with transportation issues).

### Comparative Cost-Effectiveness and Access Across Modalities

Interventional psychiatry modalities vary by the different equipment needs, treatment frequency, personnel requirement, and cost. However, when comparing across different modalities the cost-effectiveness depends not only on direct procedural and equipment expenses, but also on durability of treatment response, downstream healthcare utilization and the economic impacts of functional recovery. When considering mental parity laws in the U.S. it is also important to keep in mind that the cost of treatment in the US is overall greater than other countries, and that costs can vary substantially by region [32].

ECT while requiring anesthesia and specialized staffing, is often considered cost-effective for severe, treatment-resistant depression because of its high and rapid response rates, which can reduce hospitalization duration and associated costs [31]. Yet, ECT in the US remains largely underuse even when approximately 70%−80% of patients with MDD show significant improvement. This is mostly due to the stigma surrounding the treatment which still shapes perceptions of safety and effectiveness [30, 33, 34]. There are also other compounding reasons including bias [35], and fewer centers offering the procedure [36]. While there are not many studies focused on sociodemographic disparities in ECT, studies have reported African Americans to be underrepresented among patients treated with ECT [37–39].

TMS generally involves higher upfront equipment costs and multiple treatment sessions, but its favorable side-effect profile and sustained remission rates contribute to cost-utility over time, particularly for patients who would otherwise require prolonged pharmacotherapy. Several studies support rTMS cost-effectiveness for TRD. For example, Simpson and colleagues [40] found that TMS is a cost-effective treatment for patients who have not benefited from initial antidepressant pharmacotherapy. A recent study assessing actual healthcare utilization and costs of rTMS in the United States found that rTMS for TRD reduced health care system utilization and insurance cost over an 18-month timeframe [41]. Ongoing research is seeking to identify and optimize clinically relevant stimulation parameters to improve the effects of rTMS [42, 43].

In the case of TMS, at least according to standard non-accelerated protocols, a major practical limitation is the time commitment required. Standard TMS requires daily sessions close to an hour over several weeks. This disadvantages those with lack of transportation, stringent work arrangements, and limited social support [7, 44], and helps explained why only “0.7% of the total eligible treatment population received rTMS” [41]. Furthermore, just as with ECT, studies have found racial disparities in the use of TMS [7, 44].

Recent FDA-cleared stimulation protocols (theta burst stimulation and accelerated forms of TMS) can reduce the duration of the sessions to a few minutes, or just to a few days, with the potential to expand rTMS utilization, yet they are generally considered investigational or not covered by insurance [45]. Moreover, they might impose challenges to clinics in their operations [19].

Compared to ECT and TMS, VNS and DBS incur higher costs as they involve inpatient surgical procedures with its associated expenses (including multidisciplinary team of neurosurgeons, psychiatrists, neuropsychologist, surgical room), as well as the cost of the hardware (including electrodes, extension wires and implantable pulse generator [IPGs]). The IPG needs to be replaced periodically when its battery is close to depletion, which requires an outpatient surgical procedure.

While VNS was FDA approved in 2005 based on clinical trial data [31, 46], the Centers for Medicare and Medicaid Services (CMS) saw the data as insufficient for reimbursement. Even with data from a registry study by Aaronson et al. [26] showing long-term enhanced antidepressant effects (> 60%) compared with treatment as usual in severely ill patient population, for increased VNS coverage it was needed to show more definitive evidence of efficacy. This resulted in CMS partially supporting the RECOVER study (a prospective, multi-c**e**nter, randomized **c**ontrolled blinded trial), for which CMS agreed to reimburse participants enrolled in the study [46]. The results from the study were just recently published [47], and CMS is finalizing changes for VNS for treatment resistant depression (TRD) that hopefully will expand Medicare coverage.

In terms of disparities, there is not specific data on VNS for depression, but a 2019 study found that black patients with refractory epilepsy were less likely to receive a VNS independently of other variables [48].

While DBS remains experimental for most psychiatric conditions, Bishay and colleagues in their paper evaluating the reported costs of DBS, stress that more research should focus on comparing the cost-effectiveness of DBS and its reimbursements for different conditions [49]. A study by Kabotyanski et al. [50] conclude that DBS is both cost saving and more effective than usual treatment for treatment-resistant depression. However, for treatment-resistant OCD, Najera et al. [30] found that rechargeable DBS was more cost-effective in the treatment of treatment-resistant OCD compared to non-rechargeable DBS. Other relevant factors when considering DBS cost-effectiveness include brain regions targeted as well as stimulation parameters. Several studies are looking into optimal stimulation parameters and predictors of response [22].

In terms of access, the HDE status of DBS for OCD may contribute to its lack of equal insurance coverage, as an HDE implies an expedited FDA approval when there is enough evidence supporting a treatment for those who need it, but not enough evidence to meet traditional thresholds for non-HDE approval [27, 30]. Insurance companies use this evidence criterion to justify their lack of coverage of DBS for OCD, treating it as if it was still “experimental,” with many private insurances denying coverage of DBS for OCD [27, 51]. While the current parity law gives the opportunities to appeal denials with a transparent process for review [52], this underestimates how complex and challenging this can be for beneficiaries, who in the midst of serious illness need not only to be informed about their right to appeal but also have the ability to appeal through multiple stages. This disproportionately disadvantages individuals with lower economic and social resources. Moreover, the fact that coverage of DBS for dystonia, which is also approved under an HDE, does not seem affected in the same way [27, 51] is evidence of a clear failure in applying the Federal Parity Act.

The fact that these procedures require patients to attend several sessions or to have implanted devices which might require tune up provides ample opportunities for disparities to exist. For example, several publications have highlighted racial disparities [53–55] with patients of color being less likely to receive DBS than white patients. Other disparities are based on type of insurance [56, 57], and having higher income [56–59].

### Interventional Psychiatry Inequities in Access: Ethical and Policy Considerations

Ensuring equitable access to advanced psychiatric care is also a moral and societal imperative rooted in principles of justice, autonomy, and beneficence. Mental health conditions can profoundly affect a person’s ability to work, maintain relationships, and participate fully in civic life. Denying effective treatments to certain groups limits their life opportunities and quality of life. From a systems perspective, expanding access can reduce long-term healthcare costs by preventing hospitalization, chronic disability, and crisis-driven care. Policies that support inclusive insurance coverage, investment in community-based specialty care, workforce diversification, and culturally responsive treatment models are therefore critical. Equitable access is not merely about distributing technologies fairly, but about ensuring that all individuals have a genuine opportunity to benefit from advances in psychiatric science.

Despite the proven value and growing potential of interventional therapies in addressing mental health needs, there are disparities in access to these treatments owing to economic, cultural, and geographical factors. Even with the existence of mental health parity laws, which are intended to ensure that mental health services receive insurance coverage comparable to physical health care, insurance companies continue imposing inappropriate barriers for mental health disorders such as prior authorization requirements, complex billing rules, stringent documentation standards, step therapy policies (which require patients try and fail low-cost therapies before gaining coverage for costlier, evidence-based therapies) and utilization review processes using “medical necessity” criteria [8]. These judgments can be challenged but that effort can be burdensome for both clinicians and patients, discouraging mental health professionals from wanting to accept private health insurance, and disproportionately affecting marginalized populations with fewer economic and social resources.

Moreover, parity is not enough, as parity governs insurance benefits and does not affect services paid entirely out of pocket. There are 32 million Americans in late 2025 without any health insurance, which is a huge barrier for access to mental health services. Interventional psychiatry tools are overall costlier than first-line treatments, involving (depending on the technology) pre-operative and post-operative imaging, anesthesia, cost of each session, cost of device/medical supplies, battery replacement costs, costs of revision surgery (for DBS), surgical team fees, hospitalization cost (if applicable), as well as maintenance and adjustments that can range between $200 to $620 per visit.

There are also problems with mental health treatment access [2, 6] not addressed by parity. In 2022, among the 15.4 million adults with severe mental illness, 66.7% received mental health treatment in the past year [60], leaving 5.1 million adults without receiving any mental health treatment, 49.7% of this group perceived an unmet need for mental health treatment in the past year. Unaffordable care (58.9%) was the most cited reason among those with unmet service needs or no services received, and lack of knowledge about where to receive services (51.1%) being among the top three reasons mentioned [60]. Recent studies have also found that lack of knowledge about effectiveness [6] and awareness of these treatments are key barriers [9, 61], which are partly due to inadequate diffusion of evidence-based care into community settings.

Stigma is another important barrier to mental health access as it prevents patients from seeking treatment, or trying specific types of treatments [34]. Broader system constraints, such as provider workforce shortages, low reimbursement (contributing to high out-of-network and self-pay reliance), administrative complexity, the lack of standardized reimbursement models, inconsistent insurance coverage, and the overall investment required (both financial and logistical) [62] limit the clinical adoption and use of interventional psychiatry tools.

Finally, it needs to be remembered that the entire debate regarding mental health parity is set within a political and economic climate with demands for controlling health care costs by all payers: employers, legislators, patients, and insurers. No one will object to paying health care costs associated with therapies that save lives, that improve the quality of lives, that restore functionality, that cure or ameliorate serious diseases, or that prevent serious disease from emerging. Effectiveness matters. Cost-effectiveness matters. Therapies that yield, or are likely to yield, only marginal benefits at very high cost deserve only low priority or no priority. This is the larger problem of parity in the entire health care system. This is why we need to address the larger problem of priority-setting in health care. Not every medical therapy that can offer some medical benefit is equally worthy of funding. But for those interventions where the evidence shows high efficacy they need to be accessible to everyone who needs them, so the issue with parity in interventional psychiatry is how to ensure that those generally disadvantaged in our society are not left out of the benefits these interventions can bring to them.

## Conclusion

As interest in interventional psychiatry interventions grows—particularly given the limited efficacy and accessibility of existing treatments for chronic and treatment-resistant mental health conditions—future research must evaluate the durability of benefits and cost-effectiveness to support broader and more equitable coverage. Given constrained healthcare resources, carefully designed step-therapy approaches may be appropriate, shifting parity concerns toward inconsistent coverage policies and plan requirements rather than outright exclusion. Considering that interventional psychiatry modalities are resource-intensive, costly, and unevenly covered by insurance, it is not surprising that financial wealth and sociodemographic factors are associated with their use. Thus, more research is needed to better understand these disparities and to address them. Ultimately, achieving mental health parity will require more than insurance reform alone; it must be accompanied by investments in workforce development, training, delivery infrastructure, and education to ensure that those who truly need interventional psychiatric treatments can access them.

## Data Availability

No datasets were generated or analysed during the current study.

## Footnotes

1 In the U.S., uneven coverage is less about a single uninsured group and more about systematic differences across payors. For commercial payors (private insurers like UnitedHealthcare), deductibles and out-of-pocket costs can be high leading to financial barriers despite being insured. In the case of government payors, Medicaid specifically serves low-income individuals, but since states administer Medicaid, eligibility, benefits, and access differ significantly by state, contributing to uneven coverage nationally. Medicare covers seniors and certain younger people with disabilities, but has incomplete coverage (e.g., limited long-term care) often requiring supplemental coverage to reduce out-of-pocket costs producing underinsurance even among the insured.

2 https://www.ipconsortium.org/
